# The Psychosocial and Spiritual Components of a Good Death

**DOI:** 10.1093/geroni/igaa057.1355

**Published:** 2020-12-16

**Authors:** Quentin Maynard, Ellen Csikai

**Affiliations:** 1 University of Southern Indiana, Newburgh, Indiana, United States; 2 University of Alabama, Tuscaloosa, Alabama, United States

## Abstract

Advances in medical care led to more older adults living longer, with at least one chronic illness. Correspondingly, the disease trajectory and dying process may be prolonged, providing more time to plan for a ‘good death.’ A ‘good death’ is typically described in the literature according to medical and biological aspects, with limited explicit discussion of psychosocial and spiritual elements. The purpose of this study was to explore older adults’ perceptions of psychosocial and spiritual factors that contribute to a death they would consider ‘good’. Using descriptive qualitative research methods, 12 community-dwelling older adults in central Alabama participated in two in-depth interviews. The findings suggested a range of physical, psychological, social, and spiritual components contributed to the conception of a ‘good death’ and were often interrelated. For example, the medical treatment desired at end of life was influenced by the perceived effects on the participants’ family/social network. Reflecting on previous experiences with death, along with motivations and values, guided what participants believed could help achieve their ‘good death.’ Most realized that preparation would allow for control over their experiences at end of life. The study findings emphasized the need to consider the holistic nature of advanced planning for the end of life according to what older adults and their families perceive as important to a ‘good death’. Efforts by professionals to maximize quality of life throughout serious illness and near the end of life are essential and can be accomplished through understanding what a ‘good death’ means to older adults.

